# The Feasibility of Long-Segment Fluoroscopy-guided Percutaneous Thoracic Spine Pedicle Screw Fixation, and the Outcome at Two-year Follow-up

**DOI:** 10.5704/MOJ.1911.007

**Published:** 2019-11

**Authors:** FC Tamburrelli, A Perna, L Proietti, G Zirio, DA Santagada, M Genitiempo

**Affiliations:** 1 UOC Vertebral Surgery, IRCCS Fondazione Policlinico Universitario Agostino Gemelli, Rome, Italy; 2 Institute of Orthopedic Clinic, Università Cattolica del Sacro Cuore, Rome, Italy

**Keywords:** percutaneous, minimally invasive surgery, thoracic spine fractures, metastasis and infection, neuro-navigation

## Abstract

**Introduction:** Posterior percutaneous instrumentation may represent a challenge when multiple levels need to be instrumentated, especially when including the upper thoracic spine. The aim of the present study was to evaluate the technical feasibility and the long-term outcome of such long constructs in different surgical conditions.

**Materials and Methods:** This investigation was a retrospective cohort study which included patients who underwent thoraco-lumbar percutaneous fixations. We collected clinical, surgical and radiological data, with a minimum follow-up of 24 months. Health-related quality-of-life, residual pain, instrumentation placement, and complications were studied.

**Results:** A total of 18 procedures were enrolled, in which 182 screws were implanted, (170 positioned in thoracic and 12 in lumbar pedicles, respectively). No surgical complications or hardware failure occurred in our series, 6 out of 182 (3,2%) screws had a partial pedicle breach, without neurological impairment or need for surgical revision.

**Conclusion:** According to our results, a fully posterior percutaneous approach for long thoraco-lumbar spine instrumentation can be considered safe and reproducible, although an adequate training is strictly required.

## Introduction

Minimally invasive percutaneous approach to the thoracic spine (TS) has been carefully questioned by experts due to the risk for severe associated complications. The local anatomy, heterogeneous patient-by-patient, the limited visualization of the surgical landmarks using the fluoroscope, especially for the upper TS, represent the main surgical challenges. Furthermore, low chances for arthrodesis should be considered. Many conditions involving the TS require instrumentated surgeries, using long hardware-constructs to provide an adequate mechanical support. The risk for complications increase proportionally to the number of involved levels and prosthetic implants. Some authors reproduced their experience in lumbar minimal invasive spinal surgeries (MISS) on the TS, reporting good clinical outcomes^1-4^.

Modern intra-operative image guidance systems, specifically developed for pedicle screwing, were demonstrated to be reliable in minimising the risk of screws mis-positioning and neurologic injuries^5^. We already reported our experience in either pure percutaneous or hybrid with open techniques for TS instrumentations. Therein, the possibility for a short open approach on upper TS level was rated as safer, whenever the percutaneous technique considered risky for the aforementioned limitations. However, those cases administrated purely percutaneously were encouraging in terms of surgical and clinical outcomes. Accordingly, we designed this retrospective investigation to evaluate the safety and feasibility of pure percutaneous surgeries for TS long instrumentations.

## Materials and Methods

The present investigation consists of a retrospective analysis of radiological and clinical data of patients underwent pure percutaneous posterior (PPP) TS instrumentation at our institution, between 2012 and 2016.

Data was collected from the institutional database and imaging repository. The consensus for surgical and scientific purposes was collected from every patients at the time of surgery, according to the institutional guidelines. Institutional database, according to the time range, was screened for patients who underwent thoracic PPP instrumentation involving four or more segments. Only cases with complete clinical and radiological data, and a minimum follow-up of two years were considered for inclusion. Previous spinal surgery was considered as exclusion criteria.

**Fig. 1:**
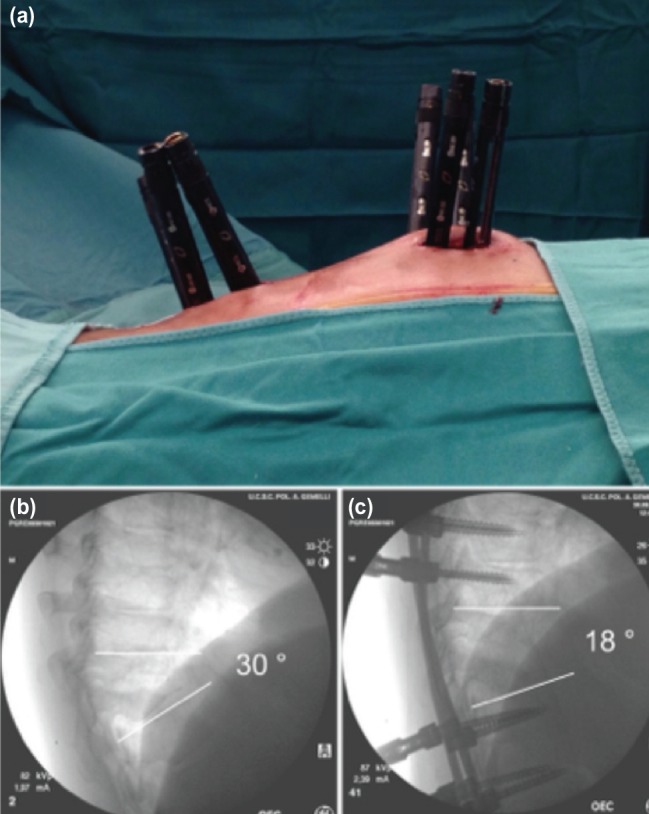
(a) Intra-operative image of percutaneous approach in patient with pyogenic spondylodiscitis T10-T11. (b) Preoperative fluoroscopic image showing high grade vertebral bodies destruction and kyphosis of the thoracic spine at T10-T11 level. (c) Fluoroscopic image after percutaneous posterior stabilisation. More than 10 degrees of correction were obtained with the posterior approach alone.

Eighteen patients (ten males, eight females) (Table I) were finally included in this case series. Two different instrumentation systems [Viper system, DePuy Synthes Spine®; and Precept, Nuvasive ®], the C-Arm fluoroscopic device and intra-operative neuro-monitoring were used. Before starting the surgical procedure a good fluoroscopic visualisation of the pedicles was obtained^5^. The entry point was identified in the supero-lateral portion of the pedicle, on the antero-posterior (AP) view. A guide wire was inserted from the entry point, through the pedicle to the vertebral body under radiographs guidance; then, the selected cannulated screw was inserted following the wire trajectory, until it reached the medial border of the pedicle in AP view. Thereafter, the screw trajectory was verified using the latero-lateral (LL) view. An adequate modeling of the rods before their positioning was pursued to achieve an adequate thoracic kyphosis, usually reproducing the native curvature of the patients (Fig. 1).

**Table I:** Details of the patients. General data, etiology, the levels affected, the levels instrumented and comorbidities. Abbreviation: NU = Non Union, AS= Ankylosing Spondylitis, BCM = Breast Cancer Metastasis, SP = Streptococcus Pyogenes, TBC = Mycobacterium tuberculosis

Pt n°	Sex	Age	Fracture	Tumor	Infection	Instrumented level	n° of screws	Other fracture reported	Comorbilities
1	M	41	T4,5,6	/	/	T2,3,8,9	8	Sternum	Lung contusion
2	M	61	T4,5	/	/	T2,3,6,7	8		Lung contusion
3	M	54	T5,6,12	/	/	T3,4,8,10,11	10		Head injury
4	M	49	T4 NU	/	/	T2,3,6,9,10	10	Ribs	Smoker
5	M	78	T5,6 AS	/	/	T4,5,7,8	8		COPD, Diabetes, Hypertension
6	M	67	T4,5 AS	/	/	T2,3,6,7,8	10		Diabetes, Heart falure
7	F	59	T4,5	/	/	T2,3,6,9,10	10	Ribs, spinosus apophysis	Lung contusion
8	F	39	T5,6 NU	/	/	T2,3,4,7,8,9	12		Smoker, Diabetes
9	F	22	T7,9,12	/	/	T6,8,10,11, L1, L2	12		Lung contusion
10	F	37	T5,6,8	/	/	T3,4,7,9,10	10		
11	F	16	T1, 2,5,6,7,8,9, L1,5	/	/	T3,4,7,8,11,12, L2	14	Sacrum, coccyx, distal humeri	Pneumotorax, splenic ropture
12	F	25	T6,7	/	/	T4,5,8,9	8		
13	M	34	/	/	T6 TBC	T4,5,7,8,9	10		Smoker
14	M	45	/	/	T4 ,5 SP	T2,3,6,7,8	10		
15	M	18	/	/	T4 TBC	T2,3,5,8,9	10		
16	M	50	/	/	T10,11 SP	T8, T9, T12, L1	8		
17	F	57	/	T6,7,MBC	/	T4,5,11,12	8		Diabetes
18	F	49	/	T4,6,10,11 MBC	/	T2,3,5,8,9,12, L1,2	16		Dead 26 month after surgery

The size of each screw was adapted to the pedicle morphology and its dimensions, according to a criteria; to be as long, as tick and high as possible (from 4.5mm to 6.5mm). The length of the screws was always 40mm in the lower segments (T12-L2) and did not exceed 35mm in the most cephalic ones (T7 or above). Fusion was not promoted in any case, according to the surgical techniques.

The Oswestry Disability Index (ODI) and Visual Analogue Scale (VAS) scores were collected to evaluate the clinical status. The hardware integrity was evaluated using standard radiographs at 1, 6, 12 and 24 months after surgery. Implant failures, presence of radiolucency or signs of subsidence around the screws, pull-out or implants loosening were investigated. 12 months after surgery, all patients underwent thin slices CT-scan with coronal and sagittal multi-planar reconstruction. The screw positioning was classified according to Youkilis’s method^6^ by three different authors; the evaluation was based on the images collected from the CT scans collected 12 months after surgery, according to the institutional follow-up protocol.

Parametric data were statistically analysed using the t-Student. The significance was established for a value of p <0.05. Data are presented as the mean and range (min-max). Dedicated SPSS statistical calculation software [SPSS Inc, Chicago, IL] was employed.

## Results

**Fig. 2:**
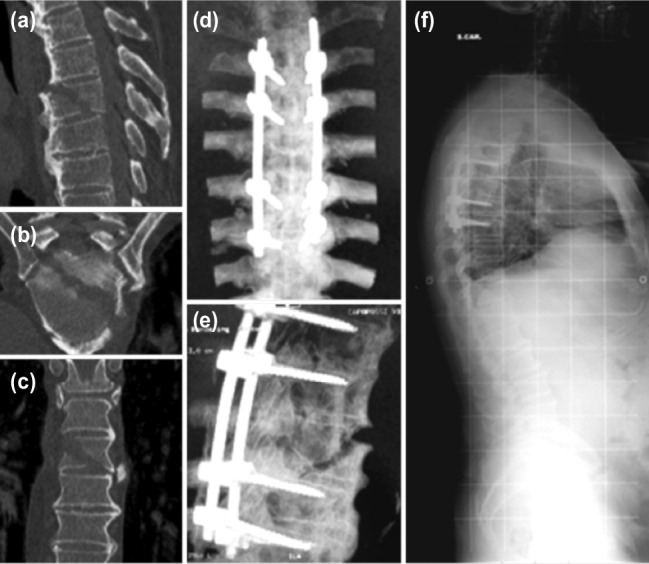
(a,b,c) CT-scan investigation in a male patient affected by ankylosing spondylitis, revealing severe T6 fracture starting from the ossified anterior longitudinal ligament and involving the disc and the vertebra. (d,e) Postoperative CT-scan 3D reconstruction showing the correct placement of the screws in T4, T5, T7 and T8. (f) Radiograph of the spine at two years follow-up, no movement or implant loosening evident.

The minimum post-operative follow-up was 24 months. The mean age was 44.5 (+/-17.2) years. Eight patients of our series had poly-trauma patients with an Amielic type A3 fractures, according to AO Spine thoracolumbar spine injury classification system7, as a consequence of major traumas. They presented with associated lesions. The most common were: ribs, spinous processes, costo-transversal joints, sacrum and coccyx fractures and, in one case, elbows bilaterally (Table I). Lung contusion with respiratory impairment was associated in four cases, head injury in one patient and splenic injury in one case. Two patients were affected by ankylosing spondylitis (AS), in one case the patient had an Amielic type B2 fracture and in the other case a Amielic B3 fracture^7^ (Fig. 2). Two patients had a non-union in a vertebral fracture occurred respectively, 8 and 13 months before. Two patients were affected by thoracic metastasis of breast cancer involving vertebral body. Four patients presented with spinal infection: in two cases were related to a pyogenic spondylodiscitis *(Streptococcus pyogenes)* and in another two case were related to *Mycobacterium tuberculosis.* The affected segment was T5 in eighteen, T6 in ten, T4 and T7 in eight each, T9 in four, and T8, T10, T11 and T12 in two cases each. More than one vertebral bodies was involved in 16 patients.

One-hundred eighty-two screws were implanted, 170 in thoracic pedicles and 12 in lumbar pedicles (Table I). The upper and lower instrumentated levels were T2 and L2, respectively. The average operating time was 134.7 minutes (min 89 - max 193).

The length of hospitalisation was of 13.4 (min 6 - max 31) days post-operatively. We did not report perioperative complications in this case series. The mean follow-up was 28.7 (min 24 - max 31) months, post-operatively. The VAS back score was 7.9 (min 5 - max 9) before surgery, 4.8 at (min 2 - max 7) at one month, 3.2 (min 1 - max 5) at three months, 1.3 (min 1 - max 4) at one year and 1.1 at two years (min 1 - max 3) (p=0.002). The “Oswestry Disability Index” score was 64.7% (min 46% - max 72%) before surgery, 36.3% (min 28% - max 42%) after 3 months, 18.4% (+/- min 12% - max 28%) after one year and 16.6% (min 12% - max 22%) after two year (p=0.01). There were no patients showed complications related to implants, such as subjective discomfort. No loosening or failure of the implant during follow-up were observed. One patient died, due to the oncological disease, after two years.

The accuracy of the thoracic screws placement was rated, according to the classification system proposed by Youkilis *et al*^6^, as optimal for 164 percutaneous screws (Grade I) and acceptable for the remaining six thoracic screws (Grade II). The 12 lumbar screws were all evaluated as optimally positioned (Grade I). No cases of iatrogenic neurological impairments were observed and no further surgical treatments were required.

## Discussion

The advantages of percutaneous pedicle screw fixation have been widely reported; reduced blood loss, less soft tissue trauma, less perioperative pain, shorter hospitalisation and earlier return to normal activities compared with open surgery procedure. Furthermore, minimally invasive spinal surgeries for instrumentation, when performed in an emergency setting, might show great potentiality for reducing the surgical invasiveness, thus allowing a faster mobilisation, which might improve the overall outcome in poly-trauma patients^8, 9^. A recent meta-analysis, conducted by Feng Tian *et al*^10^, showed that the percutaneous procedures for treating thoracic and lumbar spine fractures have better results in terms of post-operative pain, blood loss, time of operation, hospital stay; however, there were no advantages in terms of radiological outcomes and perioperative complication rates. On the other hand, some disadvantages were also reported: longer operating time, increased use of fluoroscopy, inadequate deformity correction, and lower chances for arthrodesis. Since they have been adopted as common surgical practice, minimally invasive techniques have showed great potentialities in improving clinical and surgical outcomes in spinal procedures, although their superiority in many scenarios is still questioned. Imajo *et al*^11^ reported that the complication rate, especially the postoperative infections, is significantly higher in open procedures, even more in instrumented ones.

Percutaneous instrumentations in thoracic spine have been reported as dangerous procedures, due to the complex anatomy and the limited visualisation of the bony structures, especially in upper segments. Recently, various navigation systems have been released to increase accuracy and safety, especially in deformity cases, as confirmed by Lieberman *et al*^12^.

Kakarla *et al*^13^ treated with a pure percutaneous approach six patients affected by thoracic fractures (five acute unstable thoracic fracture and one osteoporotic burst fracture), using an intra-operative Iso-C-arm fluoroscopy. Accuracy of screw placement was investigated by post-operative CT scan according to the method of Youkilis *et al*^6^. They concluded positively about the feasibility of percutaneous instrumentation for complex thoracic spinal fracture. Neuro-navigation, based on Cone Beam 3D CT scans, has also been shown as a useful tool in trauma surgeries, reducing the radiographs exposure and screws malpositioning^14^.

In a cadaveric study no significant difference in pedicle screw placement in the thoracic spine (T4-T12) was found between percutaneous fluoroscopy- guided pedicle technique and the conventional open technique^15^. A retrospective study on a large series of patients, who underwent thoracolumbar surgery, treated with 1.609 pedicle screws using CT scan three-dimensional positioning system, demonstrated the same rates of pedicle, vertebral body and facets breach between percutaneous and open procedures^16^. The screws positioned with a free-hand technique tend to perforate the medial cortex, whereas those positioned under CT navigation guidance, tend to perforate the lateral wall. Although there is no clear reason for this difference in the outcome between free hand and CT guided pedicle screwing, navigation system could contribute to increase safety, since medial misplacements are more likely providing neurological deficits^17^.

Nevertheless, Eck *et al*^18^ showed that the use of intra-operative CT-guided navigation is more useful in thoracic than in lumbar spine, in terms of accuracy.

Undoubtedly the accuracy of the navigated screw placement is higher in comparison with “free-hand” techniques, but navigation systems are often not readily available mostly for economic reasons; moreover, percutaneous pedicle screw placement in the thoracic spine, without image navigation and with a standard C-arm fluoroscope, also demonstrated to be a safe alternative^19, 20^.

The major limitations for adopting these techniques on the thoracic spine were the anatomical peculiarities of the thoracic pedicles and their difficult fluoroscopic visualisation^21^. The anatomy of the thoracic pedicles change in morphology, dimension and position in the space depending on the level. In the lower thoracic spine, the pedicles have the largest diameters even in the coronal than in the sagittal axis. They are positioned approximately in the middle of the vertebral body, appearing in fluoroscopy as an oval image impressed over the silhouette of the vertebral body. Moving above to mid thoracic spine, the pedicles become slighter and positioned in the proximal third of the vertebral body. The pedicles of the upper thoracic levels increase again in width.

Because of this, it has been shown that the smaller diameters of the mid-thoracic pedicles were the first to cause influencing breach rates^22^.

The best visualisation of the radiographic edge of the pedicles had to be checked before starting the procedure. In the proximal levels the shoulders hide the visualisation of the pedicle in lateral view; in such condition it was mandatory to obtain a perfect coronal view with the C-arm oriented according to the patient’s kyphosis. Thoracic pedicles appeared fluoroscopically as a longitudinal oval shape image, formation, overlapping the lateral wall of the vertebral body, just below the upper endplate. These margins were the percutaneous pedicle screwing landmarks. To avoid spinal canal encroachment, the screw progression into the pedicle must be fluoroscopically checked, giving a slightly medial direction without exceeding the medial border of the oval and far enough from the vertebral body superior edge, to avoid the disc space violation. Following such rules, the orthogonal-lateral view is necessary only for checking the screw length. Considering the reduced dimension of the upper thoracic vertebral bodies compared to the lower thoracic or lumbar spine, screws exceeding length of 35mm are generally not used. Diameter has usually to be chosen according with the radiographic morphology, analysed on pre-operative CT scans and checked on intra-operative fluoroscopic images.

No major complications related to wrong screw placement or their mobilisation were registered in our series. In the present series 170 screws were placed and, at CT scan evaluation, only 13 screws violated the medial margin of the pedicle for less than 2mm, without canal encroachment or neurologic complication. Equally safe results have been also reported by Park *et al* in a series of 172 percutaneous screws implanted under fluoroscopic guide checked by CT scan in the post-operative follow-up.^23^ Patients in our series showed significant pain reduction even at one month after surgery. After two years the pain was no longer affecting patient’s quality of life. The function, evaluated by the ODI, improved one month after surgery and at any subsequent follow-up visit. The post-operative nursing was favoured by the minimally invasive approach, allowing the prompt mobilisation, rapid improving of the breathing capacity, thus a shorter hospitalisation and lower health-related costs. In a recent study Zhao *et al*^24^ analysed the complications of percutaneous pedicle screw fixation in thoracolumbar and lumbar fractures. The most common complications were; guide wire ruptures, blood vessel injuries, cerebrospinal fluid leakage, screws misplacement, poor reduction, failed internal fixation, and infections. However, in the present series no specific difficulties or complication were registered applying the described technique to the upper thoracic spine, where percutaneous screw insertion would be considered less feasible and more dangerous.

Low chances for arthrodesis is usually reported as the major limitation for pure percutaneous instrumentation techniques. In our series we did not promote fusion, relying on the stability of the implant only. We think that, according to the heterogeneity of the diagnosis, our results would not allow to properly evaluate the fusion rate. Furthermore, the primary objective of this study was the evaluation of the feasibility and safety of a thoracic fully percutaneous thracic screw fixation only using fluoroscopic guidance.

## Conclusion

The fluoroscopically guided percutaneous approach to the thoracic spine, including the upper levels, using the principles of minimally invasive surgery is a safe and reproducible method of treatment in poly-trauma patients with fractures, and in those with metastatic disease and infections, involving the thoracic spine. It is a reliable alternative to the open free-hand pedicle screw fixation which relies primarily on recognisable anatomical landmarks.
